# Editorial: Heat stress: response, mitigation, and tolerance in plants

**DOI:** 10.3389/fpls.2023.1266765

**Published:** 2023-08-14

**Authors:** Ranjeet R. Kumar, Biao Jin, Nianjun Teng

**Affiliations:** ^1^ Division of Biochemistry, Indian Council of Agricultural Research (ICAR)-Indian Agricultural Research Institute, New Delhi, India; ^2^ College of Horticulture and Landscape, Yangzhou University, Yangzhou, China; ^3^ College of Horticulture, Nanjing Agricultural University, Nanjing, China

**Keywords:** heat stress, signalling molecule, stress-associated genes, transcription factor, thermotolerance, phytohormones, secondary metabolites, heat shock proteins

Heat stress is one of the major problems affecting the growth and yield of most of the agriculturally important crops. It causes oxidative bursts inside the cells, damaging the cell organelles, membranes, and leads to the denaturation/aggregation of enzymes (Kumar et al., 2023). In order to cope with the fluctuation in temperature, plants have developed various mechanisms like the expression of signalling molecules (like MAPK, CDPK, etc.), antioxidant enzymes (SOD, CAT, GPx, etc.), Heat shock transcription factors (TaHSFA6e, HSF3, etc.), heat shock proteins (sHSPs like HSP17, HSP26, etc.) and many other stress-associated genes like pyrrolyl disulphide isomerase, ABC-Transporter, universal stress proteins, etc. (Azameti et al., 2022).

This Research Topic has been meticulously organized, featuring thirteen original research articles that have been divided into two broad areas to emphasize the findings in the best possible manner: (1) Response of plants to stress/secondary metabolites or hormones, and (2) Omics approaches for the identification of stress-associated genes and proteins.

## Response of plants to stress/secondary metabolites or hormones

The intensity and duration of heat stress have severe implications for plant growth and development. Various priming agents and hormones have been reported to modulate the HS-tolerance level of the plant. Otálora et al. reported that MeJA does not affect the gas exchange, chlorophyll A concentration, and the efficiency of the photosystem under HS. The ameliorative impact of MeJA on plants can be utilized to develop thermotolerant crop with improved grain-quality.

Secondary metabolites have been reported as one of the very important metabolites that are mainly utilized by plants as protectants against biotic stresses. Xu et al. reported that monoterpenes (eucalyptol, camphor, linalool, and borneol) modulates the antioxidant enzymes activities of *Cinnamomum camphora* under HS due to the alterations in expression of the genes related to non-enzymatic and enzymatic antioxidant formation. Various exogenous treatments using phytohormones, polyamines or organic chemicals have been reported to enhance the thermotolerance level of plants. Zhang et al. reported that brassinolide and salicylic acid reduces the damage caused by the accumulation of reactive oxygen species in rice and helps in enhancing the HS-tolerance level. Volatile organic compounds have been reported to block signalling molecules and compromise the response of plants under biotic and abiotic stresses. Pavlovič et al. reported an increase in the cytoplasmic calcium level [Ca^2+^]cyt in response to diethyl ether application in *Arabidopsis thaliana*, which in turn enhances the tolerance of photosystem II by enhancing the expression of HSPs. Exogenous application of volatile organic compounds can be used as potential method to modulate the tolerance level of the plants under different stresses.

The combined effect of heat and drought has been observed to disturb many ecosystems in terms of their water and carbon budgets. Li et al. reported that dew benefits are cancelled by the heatwave due to the minor contribution of dew in leaf water. Heat-induced reductions in net ecosystem production (NEP) were intensified by the combined effect of drought stress.

Agricultural and horticultural crops are highly prone to HS, since it has very drastic effects on different biological functions like fertilization, cell growth, fruit setting, etc. Tomato, being sensitive to HS, showed a severe decrease in yield and quality under HS. Nutrient management has been used in the past to enhance the tolerance level of the plant against abiotic stresses and has been observed to be quite effective in the case of horticultural crops. Luo et al. reported that short-term sub-high temperature (SHT) stress in tomato grown under nitrogen supplement improved the photosynthetic efficiency, nitrogen metabolism, and fruit quality. Moderate nitrogen application stabilizes the photosynthesis and nitrogen efficiency in tomato under HS and can be recommended as one of the approaches to enhance the tolerance and fruit quality of the tomato plant. Similarly, Mubarok et al. developed parthenocarpic tomato mutants and reported enhanced plant adaptability and fruiting ability under HS. Development of parthenocarpic fruit can be used as one of the approaches to enhance the tolerance and fruiting ability of the horticultural crops.

## Omics approaches for the identification of stress-associated genes/proteins

With the advent of technologies, different omics approaches are used to identify novel or putative genes, stress-associated proteins, and metabolites linked with HS-tolerance, in order to understand the mechanism of HS-tolerance. Ikram et al. used the RNA-seq method to identify stress-associated genes in flowering Chinese cabbage (*Brassica campestris* L. ssp. chinensis) and identified 20,680 differentially expressed genes (DEGs) consisting of signalling molecules, transcription factors, and regulatory proteins. Phospholipase C (PLC) has been characterized as playing a very important role in signalling the plant against HS. Annum et al. reported that over-expression of PLC5 (PLC5 OE) in *Arabidopsis thaliana* protects the disintegration of chlorophyll in leaves and has well-developed root architecture under HS. This mechanism can be used in other crop plants to modulate their tolerance level under HS. Similarly, Xue et al. reported that with an increase in temperature, an upregulation of stress-associated genes like CfAPX1, CfAPX2, CfHSP11, CfHSP21, CfHSP70, CfHSFA1a, CfHSFB2a, and CfHSFB4 were observed in *Cryptomeria fortunei* under HS.

Heat stress during pollination has a severe effect on spikelet fertility and yield in rice. Mo et al. reported that sequence variations in OsGRF4 (*Oryza sativa* growth-regulating factor 4; OsGRF4^AA^) helps in escaping the microRNA miR396-mediated degradation of this gene at the mRNA level and affects proper transcriptional and splicing regulation of genes under HS, regulates carbohydrate and nitrogen metabolism, and results in compromised tolerance behaviour in rice pollen. Huang et al. identified differentially expressed genes involved in HS-adaptation of *Gracilaria bailinae* (macroalgae) using RNA-seq and observed downregulation of most of the identified DEGs. These DEGs involved in spikelet fertility can be manipulated to enhance the tolerance under HS.

Various gene regulatory networks operate inside the plant system and play important roles in multiple adaptive processes. Xu et al. reported that drought- and cold-responsive gene regulatory networks (GRNs) in *Myrica rubra* have been built according to the timing of transcription under both abiotic stresses, and have a conserved trans-regulator and a common regulatory network. Functional validation showed MrbHLH10 to mitigate abiotic stresses through the modulation of ROS scavenging.

Overall, this Research Topic provides very significant information on role of different elicitors/metabolites in modulating the HS-tolerance, putative stress-responsive genes and their regulatory networks, and nutrient management approach to enhance the tolerance of horticultural crops. There is further need to elucidate the mechanism of heat stress tolerance in plants and identification of potential markers for evaluating the diverse germplasm of crops. This will pave the way for the development of ‘climate-smart’ crops.

## Author contributions

RK: Writing – original draft. BJ: Writing – review & editing. NT: Writing – review & editing.
